# Wdr5 is essential for fetal erythropoiesis and hematopoiesis

**DOI:** 10.1186/s40164-023-00385-3

**Published:** 2023-04-15

**Authors:** Lulu Liu, Yanjia Fang, Xiaodan Ding, Weihua Zhou, Remi Terranova, Yan Zhang, He Wang

**Affiliations:** 1 Novartis Institutes for BioMedical Research, 181 Massachusetts Ave., Cambridge, MA USA; 2 Novartis Institutes for BioMedical Research, 4218 Jinke Road, Shanghai, China; 3 Department of Hematology, Shanghai General Hospital Affiliated to Shanghai Jiao Tong University, No. 650 Songjiang Road, Shanghai, China; 4 Novartis Institutes for BioMedical Research, Kohlenstrasse 44, Novartis Campus, 4056 Basel, Switzerland

**Keywords:** Wdr5, Erythropoiesis, Hematopoiesis, Hematopoietic stem cell

## Abstract

WDR5 is a highly conserved protein that performs multiple scaffolding functions in the context of chromatin. However, efforts to understand the function of WDR5 in normal tissues physiologically are quite limited so far. In our study, we explored the function of Wdr5 in erythropoiesis and hematopoiesis by using a hematopoietic-specific *Wdr5* knockout mouse model. We found that loss of *Wdr5* mediated by *Vav-iCre* leads to embryonic lethality with defective erythropoiesis. In addition, Wdr5-deficiency completely impairs the hematopoietic stem and progenitor cells function and might alter the immunophenotype of these stem cells and progenitors by decreasing c-Kit expression. Collectively, we identified the pivotal role of Wdr5 in fetal hematopoiesis and erythropoiesis as the de novo findings.

**Supplementary Information:**

The online version contains supplementary material available at 10.1186/s40164-023-00385-3.

**To the editor**,

The canonical function of WDR5 is as a core component of the MLL histone methyltransferase complexes [1]. While the function of several other subunits of MLL complexes has been well elucidated in hematopoiesis [2–12], little is known about the function of WDR5. Our study aimed to identify the role of Wdr5 in normal hematopoiesis by utilizing hematopoietic lineage specific knockout mouse model.

To investigate the function of Wdr5 in normal hematopoiesis, *Wdr5* was conditionally deleted in the hematopoietic lineage by *Vav-iCre* transgenic mice (Additional file 1: Fig. S1A). Strikingly, we did not observe any *Wdr5*^*f/f*^*, Vav-iCre* mice (referred as CKO mice) among 40 offspring from the intercross between the male *Wdr5*^*f/f*^ mice and the female *Wdr5*^*f/*+^*, Vav-iCre* mice at weaning age (Fig. 1A), which urged us to further dissect the effects at the fetal stage. Interestingly, the CKO embryos showed roughly normal morphology, but much paler body color and smaller-sized fetal livers (FLs) compared with the littermate control at E15.5 and E16.5 (Fig. 1B and Additional file 1: Fig. S1B). Consistently, the total cell number of FL from CKO was robustly decreased at E15.5 (Fig. 1C). We further dissected this at E13.5, and similar effect was observed (Additional file 1: Fig. S1C). However, the absolute cell numbers of FLs were comparable between CKO and the littermate control embryos at E12.5 (Additional file 1: Fig. S1D). These indicated that the defective erythropoiesis might occur in *Wdr5*-deficient embryos. Next, we evaluated the erythropoiesis (Additional file 1: Fig. S1E). Compared with the littermate control, CKO embryos showed an increased percentage of the immature population including S0-S2 but a decreased percentage of relative mature population S4, whereas the absolute cell numbers of all stages were reduced at E15.5 (Fig. 1D and Additional file 1: Fig. S1F). In addition, Ter119^+^ cells could be further divided into EryA/B/C based on the FSC parameter (Additional file 1: Fig. 1E). Though the absolute cell numbers of EryA/B/C were reduced, the percentage of EryA was increased and the percentage of EryB/C was decreased in CKO embryos at E15.5 (Fig. 1E and Additional file 1: Fig. S1F). Furthermore, we examined whether the terminal erythroid differentiation is impaired in Wdr5-deficient embryos by Hoechst 33342 staining. Although the percentage of the enucleated erythrocytes were comparable in CKO and the littermate control embryos, the total number of the enucleated erythrocytes was decreased robustly in CKO embryos (Fig. 1F) and the enucleated cells from CKO embryos showed a larger size compared with those from the littermate control determined by the FSC parameter (Additional file 1: Fig. S1G). Collectively, these data suggested that loss of Wdr5 results in the blockade of fetal erythropoiesis.

**Fig. 1:**
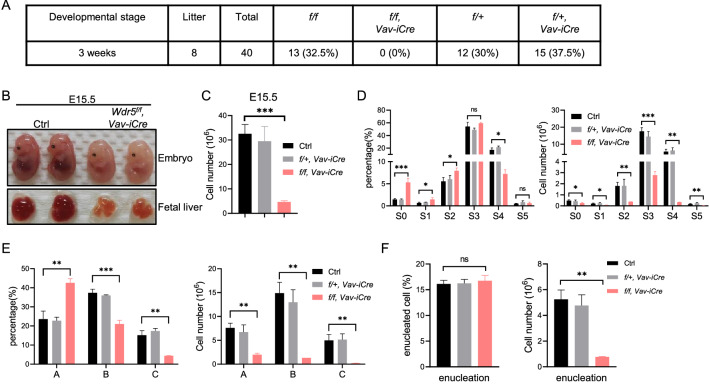
Loss of Wdr5 mediated by *Vav-iCre* leads to embryonic lethality with defective erythropoiesis. **A** The offspring from the intercross between the male *Wdr5*^*f/f*^ mice and the female *Wdr5*^*f/*+^*, Vav-iCre* at weaning age. **B** Representative photographs of the embryos (top) and the FLs (bottom) from CKO and the littermate control at E15.5. **C** The absolute cell number of FLs from CKO, heterozygous, and the littermate control embryos at E15.5 (n = 2–6 per genotype). **D** Graph showing the percentage (left) and  the absolute cell number (right) of CD71/Ter119 subsets in the FLs from CKO, heterozygous, and the littermate control embryos at E15.5 (n = 2–6 per genotype). **E** Graph showing the percentage (left) and the absolute cell number (right) of EryA/B/C subsets in the FLs from CKO, heterozygous, and the littermate control embryos at E15.5 (n = 2–6 per genotype). **F** Graph showing the percentage (left) and the absolute cell number (right) of the enucleated cells in the FLs from CKO, heterozygous, and the littermate control embryos at E15.5 (n = 2–6 per genotype). Statistical significance is indicated by ns for not statistically significant, *p < 0.05, **p < 0.01, ***p < 0.001, or ****p < 0.0001. Data are presented as mean ± SD

In addition to the defective erythropoiesis observed in the CKO embryos, we wondered whether the impaired hematopoiesis also contributed to the embryonic lethality by loss of *Wdr5*. We first examined the hematopoietic stem and progenitor cells (HSPCs) in the FLs from CKO or the littermate control embryos at E12.5. The percentage and the absolute cells number of hematopoietic progenitor cells (HPCs) (Lin^−^Sca1^−^c-Kit^+^) or LSK (Lin^−^Sca1^+^c-Kit^+^) were roughly normal in CKO embryos compared with the littermate control at E12.5 (Fig. 2A, B), indicating the initial seeding of HSPCs in the FLs was not influenced in the CKO embryos. We further examined the HSPCs in the FLs from CKO and the littermate control embryos at E13.5 and E15.5 (Fig. 2C, D). We found that the percentage and the absolute cell number of HPCs and LSK cells from FLs were significantly reduced in the CKO embryos compared with the littermate control at E13.5 (Fig. 2E). Intriguingly, the HSPCs underwent rapid expansion in the littermate control FLs but not in the CKO FLs from E13.5-E15.5 (Fig. 2E), indicating the crucial role of Wdr5 in regulating the expansion of HSPCs in FLs. Notably, the expression of c-Kit on Lin^−^ population was slightly decreased in the E12.5 CKO embryos, then further decreased in the E13.5 CKO embryos, and almost diminished in the E15.5 CKO embryos (Fig. 2A, C and D), suggesting Wdr5 might be required for the expression of c-Kit. Surprisingly, the percentage of cKit^−^Sca1^+^ population was increased in the CKO FLs at E13.5 and E15.5, so we further dissected this population. Interestingly, a certain percent of CD150^+^CD48^−^ population was found from c-Kit^−^Sca1^+^population in CKO but hardly detected in the littermate control FLs at E15.5 (Fig. 2D, G). By contrast, the CD150^+^CD48^−^ population was only found in the cKit^+^Sca1^+^ population from the control FLs but not the CKO FLs at E13.5 and E15.5, suggesting loss of Wdr5 might alter the HSPCs immunophenotype. Therefore, we performed the colony forming assay to detect the function of HSPCs. Consistently, the CKO FL cells failed to give rise to any colonies in the colony forming assay in vitro (Fig. 2H), indicating loss of Wdr5 impairs thefunction of HSPCs.

**Fig. 2:**
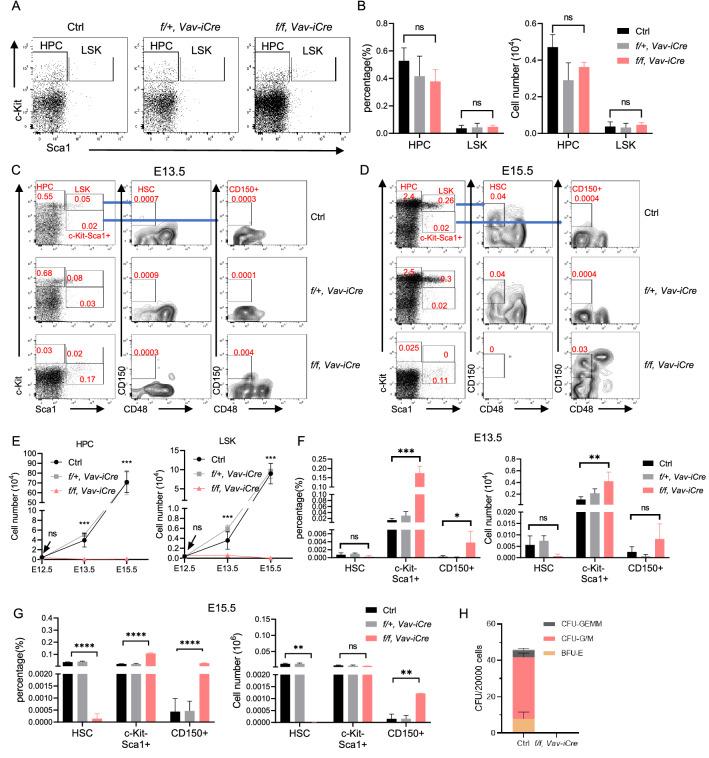
Wdr5 plays a pivotal role in the maintenance of fetal HSPCs function. **A** Representative FACS profile showing the LSK and HPC populations from CKO, heterozygous, and the littermate control embryos at E12.5. **B** Graph showing the percentage (left) and the absolute cell number (right) of the LSK and HPC populations in the FLs from CKO, heterozygous, and the littermate control embryos at E12.5 (n = 3–6 per genotype). **C** and **D** Representative FACS profiles showing the HSPCs in the FLs from CKO, heterozygous, and the littermate control embryos at E13.5 (**C**) and at E15.5 (**D**). **E** Graph showing the absolute cell number of HPC (left) and LSK (right) in the FLs from CKO, heterozygous, and the littermate control embryos at various developmental stages (n = 2–7 per genotype for each stage). **F** Graph showing the percentage (left) and the absolute cell number (right) of HSC, cKit^−^Sca1^+^, and CD150^+^CD48^−^cKit^−^Sca1^+^ (CD150^+^) subsets in the FLs from CKO, heterozygous, and the littermate control embryos at E13.5 (n = 2–4 per genotype). **G** Graph showing the percentage (left) and the absolute cell number (right) of HSC, cKit^−^Sca1^+^, and CD150^+^CD48^−^cKit^−^Sca1^+^ (CD150^+^) subsets in the FLs from CKO, heterozygous, and the littermate control embryos at E15.5 (n = 2–4 per genotype). **H** Graph showing the colony number derived from CKO and the control FLs in CFU assay. Statistical significance is indicated by ns for not statistically significant, *p < 0.05, **p < 0.01, ***p < 0.001, or ****p < 0.0001. Data are presented as mean ± SD

In conclusion, we revealed the crucial role of Wdr5 in regulating fetal erythropoiesis as well as hematopoiesis as the de novo findings, which would broaden the understanding of WDR5 function in this field. WDR5 is a very promising therapeutic target in multiple cancers including MLL-rearranged leukemia with genetic model validation and the substantial efforts have been devoted to developing the inhibitors for WDR5 [13]. Our findings for the physiological function of Wdr5 will help to elucidate the safety profile of WDR5 inhibition.

## Supplementary Information

**Additional file 1: Table S1.** List of the commercially available antibodies used in this study. **Figure S1.** Defective erythropoiesis was observed in the CKO embryos, related to Figure 1. (A) Schematic of the genetic targeting strategy to generate Wdr5 KO mice. (B) Representative photographs of the embryos (top) and the FLs (bottom) from CKO and the littermate control at E16.5. (C and D) The absolute cell number of FLs from CKO, heterozygous, and the littermate control embryos at E13.5 (C) and E12.5 (D) (n=2-7 per genotype for each stage). (E) Strategy to analyze fetal erythropoiesis. (F) Representative FACS profile showing the various developmental stages of erythrocytes in the FLs from CKO, heterozygous, and the littermate control embryos at E15.5. (G) Representative FACS profiles to analyze enucleation. Statistical significance is indicated by ns for not statistically significant, *p<0.05, **p<0.01, ***p<0.001, or ****p<0.0001. Data are presented as mean ± SD.

## Data Availability

Materials and original data generated in this study are available from the corresponding authors on the reasonable request.

## Abbreviations

KO: Knockout
FL: Fetal liver
HSPCs: Hematopoietic stem and progenitor cells
HPC: Hematopoietic progenitor cells
