# Supplementary material for: Wdr5 is essential for fetal erythropoiesis and hematopoiesis
Source: Exp Hematol Oncol. 2023 Apr 15;12:39. doi: 10.1186/s40164-023-00385-3 (PMC10105386; doi:10.1186/s40164-023-00385-3)
Supplement: Supplementary file 1 — Additional file 1: Table S1. List of the commercially available antibodies used in this study. Figure S1. Defective erythropoiesis was observed in the CKO embryos, related to Figure 1. (A) Schematic of the genetic targeting strategy to generate Wdr5 KO mice. (B) Representative photographs of the embryos (top) and the FLs (bottom) from CKO and the littermate control at E16.5. (C and D) The absolute cell number of FLs from CKO, heterozygous, and the littermate control embryos at E13.5 (C) and E12.5 (D) (n=2-7 per genotype for each stage). (E) Strategy to analyze fetal erythropoiesis. (F) Representative FACS profile showing the various developmental stages of erythrocytes in the FLs from CKO, heterozygous, and the littermate control embryos at E15.5. (G) Representative FACS profiles to analyze enucleation. Statistical significance is indicated by ns for not statistically significant, *p<0.05, **p<0.01, ***p<0.001, or ****p<0.0001. Data are presented as mean ± SD. [file 40164_2023_385_MOESM1_ESM.docx]

Additional file

**Wdr5 is essential for fetal erythropoiesis and hematopoiesis**

Lulu Liu^1^, Yanjia Fang^2^, Xiaodan Ding^3^, Weihua Zhou^1^, Remi Terranova^4^, Yan Zhang^3*^, He Wang^1,2^*

1. Novartis Institutes for BioMedical Research, 181 Massachusetts Ave., Cambridge, MA, USA.

2. Novartis Institutes for BioMedical Research, 4218 Jinke Road, Shanghai, China.

3. Department of Hematology, Shanghai General Hospital affiliated to Shanghai Jiao Tong University, No. 650 Songjiang Road, Shanghai, China.

4. Novartis Institutes for BioMedical Research, Kohlenstrasse 44, Novartis Campus CH-4056, Basel, Switzerland.

***Co-corresponding authors**:

He Wang, email: [he.wang@novartis.com](mailto:he.wang@novartis.com), Novartis Institutes for BioMedical Research, 181 Massachusetts Ave., Cambridge, MA, USA.

Yan Zhang, email: [yan.zhang19510@shgh.cn](mailto:yan.zhang19510@shgh.cn), Department of Hematology, Shanghai General Hospital affiliated to Shanghai Jiao Tong University, No. 650 Songjiang Road, Shanghai, China.

**Methods**

**Mice**

The *Wdr5^f/f^* mice line was generated in Gempharmatech. In brief, exon 3 to exon 8 of *Wdr5* endogenous locus were flanked with *loxP*. *Wdr5^f/f^* mice were further crossed with heterozygous *Vav-iCre* transgenic mice to generate *Wdr5^f/+^, Vav-iCre* mice. Then, *Wdr5^f/+^, Vav-iCre* mice were crossed with *Wdr5^f/f^* mice to obtain the indicated embryos used in this study. Genotyping and gene deletion efficiency were performed by polymerase chain reaction (PCR) using primers specific for wt (275 bp), flox (374 bp), and ko (466 bp) allele. Primers used here, 709865-Wdr5-KO-tR1: ATCAACACAGAGCATCAACACGC. mWdr5-check-s: TCTCTGCCCAGTGAGGTATGC. mWdr5-check-as: TTTGGTATGGTGGTGCATGTC. All animal protocols were reviewed and approved by the Institutional Animal Care and Use Committee (IACUC) in China Novartis Institutes for BioMedical Research Co., Ltd. and the IACUC of Shanghai General Hospital, China.

**Flow cytometry**

FL cells were isolated and passed through a 40-µm nylon cell strainer (BD Biosciences) and stained with antibodies for 20 minutes on ice in PBS supplemented with 2% FBS. Dead cells were discarded from analysis by 4,6-diamino-2-phenylindole (DAPI) (Molecular Probes). All the antibodies used in the experiments are listed in Additional file 1: Table S1. Flow cytometric analysis was performed on Fortessa or Canto II (BD Biosciences). Data were analyzed by FlowJo software (Tree Star, Ashland, OR).

**FL cell counting**

Embryos were collected from female mice at days 12.5 to 15.5 of pregnancy, and the FLs dissected from each embryo were removed into 1 mL PBS supplemented with 2% FBS. To obtain single cells, the FL were pipetted by 1 mL pipette gently and passed through a 40-µm nylon cell strainer (BD Biosciences). Then, the number of single cell suspension with or without dilution was counted by Vi-Cell (Beckman).

**Colony forming unit (CFU)**

CFU was performed according to the manufacturer’s instruction (Stemcell technologies). Regarding the CFU of FL cells, 20 000 FL cells isolated from CKO or littermate control embryos were seeded into M3434 (Stemcell technologies) in triplicates, then the colony number was counted 10 days post-seeding. Regarding the CFU of adult HSPCs, 3000 cells treated with or without 4-OHT were seed into M3434 (Stemcell technologies) in triplicates, then the colony number was counted 14 days post-seeding.

# Statistical analysis

The significance of differences was determined with two-tailed paired Student’s t tests by using Prism (GraphPad Software). Statistical significance is indicated by *p<0.05, **p<0.01, ***p<0.001, or ****p<0.0001. Data are presented as mean ± SD.

**Table S1: List of commercially available antibodies used in this study.**

| *Antibodies* | *Cat. No.* | *Company* |
| --- | --- | --- |
| Anti-Mouse-CD11b biotin | 13-0112 | eBioscience |
| Anti-Mouse-CD3e biotin | 13-0031 | eBioscience |
| Anti-Mouse-CD45R biotin | 13-0452 | eBioscience |
| Anti-Mouse-Ter119 biotin | 13-5921 | eBioscience |
| Anti-Mouse-CD8 biotin | 13-0081 | eBioscience |
| Anti-Mouse-CD4 biotin | 13-0042 | eBioscience |
| Anti-Mouse-CD5 biotin | 13-0051 | eBioscience |
| Anti-Mouse-Gr-1 biotin | 13-5931 | eBioscience |
| Anti-Mouse-Sca1 APC | 17-5981 | eBioscience |
| [Anti-Mouse CD117 (c-Kit) APC-eFluor® 780](http://www.ebioscience.com/mouse-cd117-c-kit-antibody-apc-efluor-780-ack2.htm) | 47-1172 | eBioscience |
| Anti-Mouse CD150-PE | 115903 | Biolegend |
| Anti-Mouse CD48-BV510 | 563536 | BD |
| Anti-Mouse-CD71 FITC | 11-0711 | eBioscience |
| Anti-Mouse-Ter119 PE | 12-5921 | eBioscience |


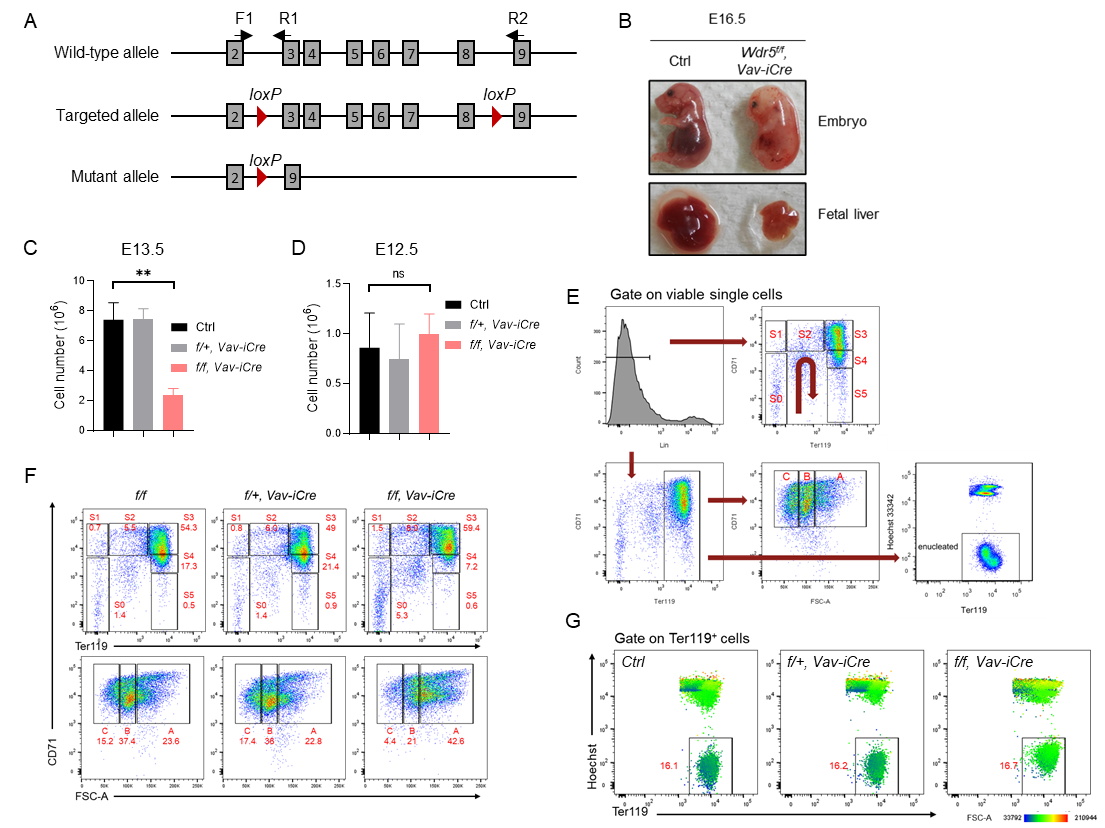
 **Figure S1. Defective erythropoiesis was observed in CKO embryos, related to Figure 1.** (A) Schematic of genetic targeting strategy to generate *Wdr5* KO mice. (B) Representative photographs of embryos (top) and FLs (bottom) from CKO and littermate control at E16.5. (C and D) Absolute cell number of FLs from CKO, heterozygous, and littermate control embryos at E13.5 (C) and E12.5 (D) (n=2-7 per genotype for each stage). (E) Strategy to analyze fetal erythropoiesis. (F) Representative FACS profile showing the various developmental stages of erythrocytes in the FLs from CKO, heterozygous, and littermate control embryos at E15.5. (G) Representative FACS profiles to analyze enucleation. Statistical significance is indicated by *p<0.05, **p<0.01, ***p<0.001, or ****p<0.0001. Data are presented as mean ± SD.
